# Supplementary figures and images for: Development and External Validation of a Nomogram Predicting Early Recurrence of Gallbladder Cancer Using Preoperatively Available Prognosticators: A Korean Multicenter Retrospective Study
Source: Cancers (Basel). 2025 Apr 26;17(9):1450. doi: 10.3390/cancers17091450 (PMC12071069; doi:10.3390/cancers17091450)

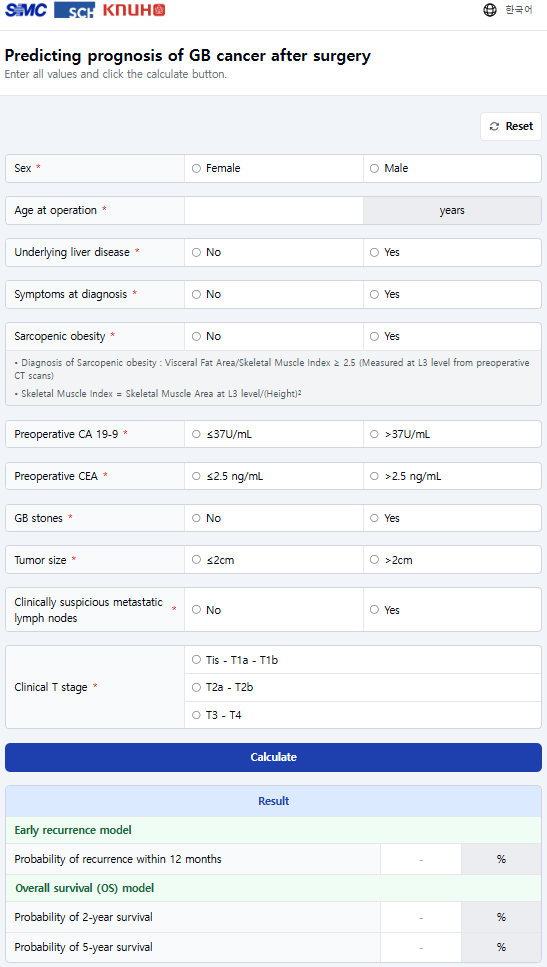

Supplement: Supplementary file 1 [file cancers-17-01450-s001.zip › cancers-3597428-supplementary.png]
